# Development and Proof-of-Concept Evaluation of a Sensory Science-Based Model for Product Development of Vegetable-Based Products for Children

**DOI:** 10.3390/foods11010096

**Published:** 2021-12-30

**Authors:** Astrid A. M. Poelman, Jessica E. Heffernan, Maeva Cochet-Broch, Janne Beelen

**Affiliations:** 1 Sensory and Consumer Science, CSIRO Agriculture and Food, North Ryde, NSW 2113, Australia; heffernan.j89@gmail.com (J.E.H.); maeva.broch@gmail.com (M.C.-B.); janne.beelen@csiro.au (J.B.); 2 CSIRO Health & Biosecurity, North Ryde, NSW 2113, Australia

**Keywords:** children, vegetables, product development, theoretical model, sensory, extrinsic product properties

## Abstract

Children’s vegetable intake is too low, and a key barrier to the inadequate intake is low acceptance. To facilitate successful development of new vegetable-based products for children, a sensory science approach to product development has been taken. A new theoretical model is proposed, the CAMPOV model: Children’s Acceptance Model for Product development of Vegetables. The model is informed by scientific literature and considers biological, psychological, and situational, and intrinsic and extrinsic product factors relevant to children’s acceptance of vegetables, with a focus on modifiable factors at the product level. Simultaneously, 14 new vegetable-based product concepts for children were developed and evaluated through focus groups with 5–8-year-olds (*n* = 36) as a proof-of-concept evaluation of the model. Children had high interest in six of the concepts. Factors identified from the literature that positively associated with the children’s interest in the concepts included bright colours, bite-sized pieces, good taste, fun eating experience, and familiarity. The CAMPOV model and proof-of-concept evaluation results can guide further sensory and consumer research to increase children’s acceptance of food products containing vegetables, which will in turn provide further insights into the validity of the model. The food industry can use the model as a framework for development of new products for children with high sensory appeal.

## 1. Introduction

Children are an important target group for the food industry, as they are not only current consumers of products, but they increasingly have the power to influence family decisions and are future grocery buyers [1]. Food promotions have a direct effect on children’s preferences, purchase behaviour, and consumption patterns, but current marketing practice predominantly promotes low-nutrient-dense foods and beverages [2,3].

The taste and texture of food are key drivers of consumption amongst children [4]. When comparing the key taste properties of vegetables to those of other core food groups, it was found that all other core foods possess taste qualities that are either innately liked or acquired very early in life [5]. Vegetables, on the other hand, do not contain such positive drivers of liking as a whole category. Rather, they contain a driver of dislike: bitter taste. Thus, the sensory properties of many vegetables do not appeal to our innate likes, and often need some form of transformation to appeal to children or need to be learned. Most children do not meet the recommended daily intake of vegetables for optimum health [6,7]. Only 5% of Australian children meet their daily, age-dependent recommended vegetable intake of 2.5–5.5 servings/day, with one serving being 75 g of vegetables [6], and the average vegetable intake of 11-year-old children across nine European countries was only 86 g [7]. Thus, novel targeted solutions to increase vegetable intake are needed.

Currently, there are not many vegetable products marketed to children [8], and food manufacturers may lack specific knowledge on how to make vegetable-based products appealing to children. A scientific framework to help guide successful product development of vegetable-based products with high appeal for children may help close this gap.

The aim of this study was to develop an evidence-informed, sensory-based model to guide product development of vegetable-based products for children, and to qualitatively evaluate children’s interest in newly developed vegetable-based concepts designed in accordance with these principles.

We first present the results from the literature review, then the proposed Children’s Acceptance Model for Product development Of Vegetables (CAMPOV), and finally, the development and qualitative evaluation of a range of new vegetable-based product concepts.

## 2. Development of the Theoretical Model

### 2.1. Materials and Methods

The Children’s Acceptance Model for Product development Of Vegetables (CAMPOV) was developed based on insights into children’s sensory preferences, knowledge of children’s vegetable acceptance, and food preference development theories. Five main factors were identified from Mojet’s proposed model of essential factors that influence eating and drinking behaviour, and food choice [9]: the intrinsic product properties (e.g., sensory modalities like appearance, taste, texture and interactions as well as dynamic contrast and complexity), extrinsic product properties (e.g. claims, brand, labelling and packaging), as well as children’s biological/physiological characteristics (e.g. age, physical condition, sensory acuity), psychological characteristics (e.g. memory, previous experiences, learning, neophobia) and situational factors (e.g. parents and peers). The key focus of the CAMPOV model is on modifiable factors at the product level.

A literature search was undertaken to support the identification of relevant intrinsic and extrinsic attributes for the model, and specifically investigated the role of these properties on increasing children’s acceptance and willingness to eat vegetables. The literature search was conducted using Web of Science and Google Scholar. A search was conducted using combinations of sensory properties (e.g., appearance, taste, texture), extrinsic properties (e.g., packaging, claims); and “vegetables”, “children”, “acceptance”, and “willingness to eat” as search terms. Bibliographies of relevant articles were screened for other relevant articles. As a target group, children aged between 2 and 10 years were included. Studies were excluded if they targeted clinical populations, individual case studies, and children with specific medical conditions, including clinically obese children and malnourished children.

### 2.2. Results

#### 2.2.1. Literature Review: Intrinsic Product Properties

This section reviews the evidence on the role sensory attributes in each modality play in children’s acceptance of vegetables.

##### Appearance

Four- to five-year-old children categorised their likes and dislikes for fruit and vegetables on appearance attributes, whereas 11–12-year-olds used taste as the basis for categorisation [10]. Using focus groups, children were found to prefer small, brightly coloured vegetables over large, dark green (leafy) vegetables [11]. Another study found that it was not just the colour itself, but rather familiarity with the colour that affected children’s evaluations, with expected liking being higher for atypically coloured vegetables (e.g., green cauliflower) than for their typically coloured counterparts [12]. A further study, using photos of different numbers of vegetables and fruit on a plate, showed that children (5–12 years) preferred a larger variety of colours (approximately six) on their plates [13].

In terms of shape and size, a preference for smaller over larger vegetables was found, although size was not systematically varied in this study [11]. Experimental research showed 9–12-year-old children liked vegetables more when cut into star shapes than when cut into chunks, slices, or sticks, and at the same time, slices and sticks were preferred over chunks [14]. However, no difference in liking between diced and whole carrot was found [15]. Visual enhancement of vegetables that aimed to retain the integrity of vegetable flavour as much as possible, improved 7–10-year-old children’s willingness to try disliked vegetables [16].

Studies examining the effect of preparation on acceptance found that uniform appearance [17] and original colour intensity [18] positively contributed to liking, and browned colour [17,18] relating to baking/frying was negatively related to liking.

##### Taste

There is ample evidence that children dislike bitter-tasting vegetables [10,11,19,20,21]. In addition, sweet vegetables are preferred over non-sweet vegetables [11,19], and the lack of a sweet taste was mentioned as the reason for low vegetable acceptance [22]. Preparation methods that enhanced a sweet taste and decreased a bitter taste were associated with higher liking [18]. Condiments can considerably change the taste of vegetables, partly due to perceptual interaction; for example, salt is known to mask bitterness [23]. Adding 0.6% salt increased the intake of green beans in toddlers [24]. Adding a sweet tastant to cucumber and green capsicum purees increased acceptance, whereas adding a sour tastant did not [25]. Using flavour–flavour learning (FFL), acceptance of a novel artichoke puree increased in 2–3-year-old children when repeatedly exposed to a sweetened artichoke puree, compared with an unsweetened puree [26]. In a study with a similar design, no difference in liking was found for salsify puree with 0.5% added salt (taste modification), or 0.2% salt plus 0.02% nutmeg (flavour modification) [27].

##### Flavour/Aroma

The strong flavour of vegetables has been mentioned by several authors as a reason for dislike or low acceptance of vegetables [11,28,29], although one study found that typical vegetable flavour positively contributed to liking [17]. Browned odour and flavour, associated with baking/frying, negatively affected children’s vegetable acceptance [12]. Boiled *Brassica* vegetables were less intense in flavour than texture-matched steamed vegetables, due to leaching out of water-soluble, flavour-active compounds, although children’s acceptance was not higher [30].

Early focus-group work by Baranowski et al. [31] found that children liked vegetables most when flavourings were added, such as raw vegetables served with a dip, or cooked vegetables served with butter or sauce, and similar findings were reported in another study by the same group [32]. Additionally, serving a plain and a herb-flavoured reduced-fat dip with vegetables improved vegetable acceptance in pre-schoolers [33]. A further study found that salt- and fat-containing dips did not increase liking, but increased intake in bitter-sensitive children [34], with similar results found for carrots to which three different herb/spice blends were added [35]. Additionally, when asked if they would be more interested in eating vegetables when served with a dip, most children indicated that they probably or definitely would be [14].

Use of mixed dishes has been suggested as a potential strategy to increase children’s acceptance of vegetables, as it may mask disliked flavour properties, whilst retaining liked textural and flavour characteristics [36]. Vegetables were frequently consumed by children in mixed dishes and their acceptance was generally good [37].

##### Texture

Some contradictory findings have been reported for texture preferences for vegetables. A preference for crunchy and dislike for “smooshie” were reported by Baranowski et al. [31]. Using photographs of vegetables in two comparable groups, children preferred hard and crunchy vegetables in one study [36], and soft and juicy vegetables in the other study [11]. Using a repertory grid method, Baxter et al. (1998) found that children preferred raw vegetables to cooked [36], which was also noted by Szczesniak [38], and a distinction between preference for raw and cooked vegetables was also used to model differences in vegetable intake in children [39]. It has further been observed that certain preparation methods were particularly associated with dislikes of certain textural properties [36]. Preparation studies found a dislike for granular [17] and tough [18] textures.

##### Sound

Children’s willingness to taste vegetables as a factor of exposure to vegetable sounds was studied by Dazeley et al. (2015) [40]. Vegetable sounds were made by manipulating the vegetable with the hands, i.e., squeezing or snapping the vegetable, and not actually putting it in the mouth, and experiencing sound through eating. Children were more willing to taste and/or touch the vegetables that they had previously been exposed to during the exposure phase of the study. To our knowledge, no studies have yet been conducted that investigate the effect of sound in-mouth for vegetable acceptance.

 

In summary, the literature review on intrinsic properties provides evidence that colour, shape, and size can influence children’s acceptance of vegetables, that children have a preference for sweeter-tasting vegetables and avoid bitter-tasting vegetables, and that flavour enhancement can increase their vegetable acceptance. Other than a preference for raw over cooked vegetables, with some further studies indicating that a crunchy texture may be preferred, evidence on the role of texture is scarce.

#### 2.2.2. Literature Review: Extrinsic Product Properties

##### Labelling/Names

The effect of a food-labelling strategy was studied, using familiar and unfamiliar versions of a dish under different labelling conditions. When no further information was provided, children chose the familiar significantly more often than the unfamiliar version of the dish. The addition of a descriptive label, whether a basic (“new dish”) or model-related (“with special mix for super heroes”) label, led to an increased frequency of choice for the new vegetable dish for carrots only, and not for broccoli [41].

##### Characters

A systematic review undertaken in 2015 on the influence of food and entertainment company mascots/characters on diet-related cognitive, behavioural, and health outcomes for children under 12, found that cartoon media characters positively influenced willingness to try a vegetable, compared with no character association, and suggested that cartoon characters can positively influence vegetable intake [42]. Other studies have corroborated this finding. Children were significantly more likely to select a vegetable when presented with an animal cartoon character [43], and children who played a videogame in which a branded character ate a healthy (fruit or vegetable) snack were more likely to eat healthy snacks themselves than when the character ate an unhealthy snack [44]. TV commercials using a newly developed cartoon character (“Reggie Veggie”) increased pre-schoolers’ preferences for vegetables [45]. Daily exposure to branded vegetable characters (vegetables with human characteristics, such as arms, legs, a mouth, and superhuman strength) through a vinyl banner around the salad bar, with or without television segments, positively affected vegetable selection from a salad bar among primary school children [46]. A further study consisting of various experiments to elucidate the specific mechanisms around cartoon-character efficacy showed that cartoon characters were effective in between-category choices, but not when comparing indulgent foods and vegetables [47]. This study showed that although cartoon characters influenced willingness to try, they did not influence liking or consumption of vegetables [47]. Further, it has been found that bimodal (audio-visual) placements were more effective in children aged 8–11 than unimodal (visual) placements in a TV show to encourage children’s fruit and vegetable consumption [48]. 

##### Visual Appeal of Packaging

There is limited information available on the visual appeal to children of packaging associated with vegetables. One study examined children’s liking for healthy foods or food images displayed as cartoons, drawings, or photos. Results showed that children liked cartoon images the most, but were more inclined to consume foods represented as photos [49]. A further study used a behavioural marketing approach in which attractive packaging aimed at 4–5-year-olds was developed, consisting of a fun and colourful design, including use of characters and incentives (stickers) within the packaging. This packaging increased consumption of vegetable snacks, compared to a non-branded control group, and interestingly, this effect was sustained after the intervention, even in the absence of the new packaging [50]. Further, the use of packaging stickers increased selection of vegetable snack boxes in school canteens [51]. Although not strictly packaging, picture books displaying pictures of vegetables have been shown to positively affect vegetable acceptance [52,53].

##### Claims

It has been shown that, for younger children, health messages need to be avoided, as these negatively impact children’s acceptance of foods [54], but studies specifically focused on vegetables are scarce. One study investigated the effect of a taste (“tastes good”) and health (“super healthy”) claim in combination with a character, which was either congruent or incongruent in shape with the target vegetable. Compared with a control condition, there was no effect of either the taste or health claim on the willingness to eat the vegetable [55]. One study investigated the use of positive vs. negative messaging in a picture book on vegetable selection [52]. In the negative-message book, the main character hated kohlrabi and frequently repeated the statement, “at least I didn’t have to eat kohlrabi!” In the positive-message book, the main character loved kohlrabi and frequently repeated the phrase, “almost as good as kohlrabi!” Additionally, kohlrabi was depicted or mentioned on every page. More children in the positive-message group chose to eat kohlrabi than either the negative-messaging or control groups [52]. 

In summary, the literature review on extrinsic properties provides good evidence on the positive role of characters on children’s acceptance of vegetables. There is some evidence on the role of labelling/names, claims, and visual appeal of packaging, although research is relatively scarce.

#### 2.2.3. Biological and Psychological Factors

##### Biological Factors

Children’s perception of foods is different from adults. They are more sensitive to a bitter taste and less sensitive to a sweet taste than adults [20]. Further, children are born with an innate liking for a sweet taste and dislike for a bitter taste [56]. Together, this means they prefer higher sweetness levels and reject bitterness levels earlier than adults [20,21]. Children acquire a liking for salty and energy-dense foods very early in life, due to positive post-ingestive feedback from fat [20].

Children also differ from adults from a texture perspective. There are significant changes in oral musculature and dentition status as children age [38]. Gradually, they develop the muscles in their mouths to move food around and the force to chew foods, which means that, over time, they develop a better ability to prepare a bolus in their mouths safe for swallowing [38]. These physiological differences lead to rejection of difficult-to-manipulate textures by young children [57], such as foods with textural contrast (e.g., juice with fibre) and slippery foods (e.g., mushrooms) [38].

##### Psychological and Situational Factors

Psychological factors are very important for food acceptance. Most of our food preferences are learned [9,20]. A recently published umbrella review (which is a systematic review of systematic reviews) based on 11 systematic reviews, which together covered 85 primary studies, has found solid evidence that familiarisation through repeated exposure to a single, and to a variety of, vegetables increases vegetable acceptance and intake in children under five years old [58]. Several reviews have shown that repeated exposure is also effective in increasing vegetable acceptance and intake in primary school children [59,60].

The abovementioned umbrella review further found support for flavour–flavour-learning as an associative conditioning mechanism, although this was not more effective than repeated exposure on its own [58]. Further emerging evidence was found for other (associative) learning mechanisms on vegetable willingness-to-try and acceptance, including the role of parental role modelling, the use of non-food rewards, and vegetable-based story books [58]. In addition, peers influence children’s acceptance for vegetables [61,62]. There are many further psychological and situational factors that can influence children’s acceptance of vegetables; however, with the focus of the current research on modifiable factors at a product level, a full review of these factors is beyond the scope of this study.

### 2.3. CAMPOV Model

The proposed theoretical Children’s Acceptance Model for Product development Of Vegetables (CAMPOV model) considers the results from the literature review on intrinsic and extrinsic attributes relevant to children’s acceptance of vegetables, and biological and psychological factors related to the child population. It considers three factors explicitly: intrinsic properties, extrinsic properties, and psychological factors (Table 1). The biological factors, and part of the psychological factors, are not explicitly mentioned in the model, as they are not modifiable and cannot be specifically addressed by product developers. However, they do express themselves through the modifiable attributes (e.g., the lack of textural contrast through physiological differences in ability to safely prepare food for swallowing).

## 3. Development and Evaluation of New Vegetable-Based Concept Ideas

### 3.1. Materials and Methods

#### 3.1.1. Product Mapping

To support the development of vegetable concept ideas, a product-mapping exercise of current, commonly available food products targeted at preschool and primary school-aged children in Australian retail was undertaken. This included a broad selection of popular products for children (e.g., confectionary items, chocolate treats, savoury snacks). In addition, an extensive search of vegetable-based products specifically marketed for children was undertaken (March 2019) at all major retailers and a main greengrocer (Harris Farm Markets). All products were assessed by a team of sensory, consumer, and food scientists (*n* = 5). During this process, products were divided into their categories (e.g., savoury snacks, vegetable-based products), and all team members individually identified intrinsic (e.g., sweet taste, sour taste, colourful) and extrinsic (e.g., bright packaging, characters, fun names) product features for the categories and individual products, using sticky notes. Once individuals had completed their individual contributions, sticky notes were clustered under the appropriate modality/attribute and discussed. Through a consensus procedure, this process aimed to identify the key intrinsic and extrinsic properties of popular products aimed at children, currently on the Australian market.

Intrinsic attributes identified in popular children’s products were:

- Appearance: bright/vibrant colours, multiple different colours in a pack, shiny, fun shapes, different shapes in one pack, shape supports name (e.g., dairy-flavoured candy in the form of a milk bottle called “Milk Bottles”), long shapes, bite size, variability in colour/size/ fillings in one pack, multiple layers (e.g., topping)
- Taste: sweet, acidic/sour, savoury
- Texture: crunchy/crispy, chewy, melting, sticky, texture contrast (e.g., hard shell with soft inside, hard shell with crunchy inside)
- Other: fizzy sensation, fun to play with

Extrinsic attributes identified in popular children’s products were:

- Packaging: bright, festive/party, characters on pack, familiarity of concepts
- Packaging size: individual pack size, easy portions, practicality
- Characters: use of different characters (cartoon, imaginary, animal)
- Names/claims: fun names, sensory claims (e.g., “Max sour: super sour, then sweet”)

Very few products containing vegetables directly marketed at children were identified on the Australian market (*n* = 17). The packaging of these had in common that they included pictures of children on the front of the packs, packaging was see-through, and bright colours were used.

#### 3.1.2. Concept Generation

A project team of sensory scientists and food technologists (*n* = 5) held a brainstorming session to come up with concepts, deriving inspiration from the insights into factors influencing children’s acceptance of vegetables from the literature review, the product mapping of popular children’s products, as well as current trends in the food industry. A range of 10 initial ideas targeting 5 to 8-year-olds was compiled. Further sessions were held to generate more ideas and fine-tune these. The focus was directed towards concepts that used fresh vegetables and processed foods with vegetables. Each idea was further discussed and developed into full concepts through various iterations. The concepts that were generated covered all eating occasions for children (main meals and snacks) and considered the eating environment (at home, school, outdoors), and the Australian eating culture. The list also included ideas fitting with current trends in the food industry, such as meal kits.

**Figure 1:**
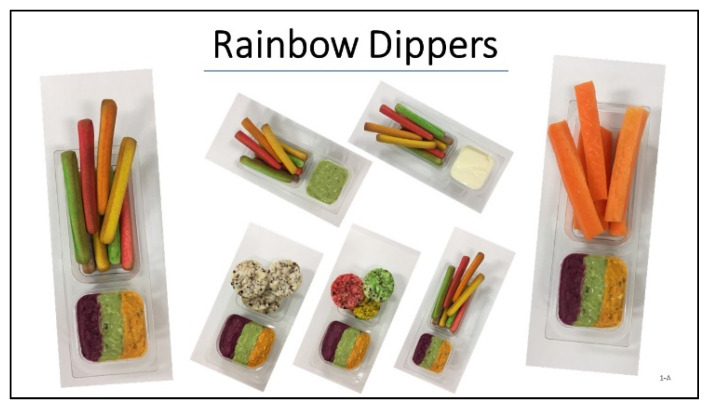
Example of concept presentation for evaluation with children.

Each potential concept was mapped against factors identified from the literature review, as well as its potential to provide meaningful increases in children’s vegetable intake. Through this process, several concepts were crossed off the list, as they did not provide enough vegetables or did not meet enough criteria of the proposed CAMPOV model, resulting in 24 concepts. Each concept was then presented in a systematic way, so they communicated the name, description with main features, and pictures for visualisation. These 24 concepts were discussed with parents of 5 to 8-year-olds in individual interviews (see Supplementary Material Part 1, including Figure S1 and Table S1 for more information). The concepts were reviewed to select the ones to include in the qualitative study with children, as due to their limited attention span, not all concepts could be evaluated with children. This selection was based on the feedback from parents on the concepts, and evaluation of the concepts by the project team for potential opportunity (weighing potential success with children and technological potential to achieve the desired sensory properties). In some cases, concepts were slightly changed, or elements from two concepts were combined. In total, 14 concepts were then evaluated with children in focus groups (Table 2). The visual presentation of concepts was adapted from what was presented to parents to account for the new target audience. In doing this, all text was removed, new pictures were sought, and titles/concept names were modified where parents commented that the concept ideas could be conveyed better. Concepts were presented on A3 sheets and showed the product/concept name with pictures; see, for example, the Rainbow Dippers concept (Figure 1).

#### 3.1.3. Focus Group Evaluation of Concepts with Children

A qualitative study was undertaken by conducting semi-structured focus groups with children (October 2019). The aim of this study was to evaluate children’s appeal of the developed vegetable-based concepts, and to provide a proof-of-concept evaluation of the CAMPOV model.

##### Participants

Children aged between 5 and 8 years were recruited through their parents to participate in the study (see Supplementary Material Part 2 for more information). Each focus group was conducted with 4–6 children to maximize the group dynamic [63]. To ensure that everyone felt comfortable in the discussion (being among their peers), children were grouped by age (5 and 6-year-olds together and 7 and 8-year-olds together) [64] and split between vegetable-likers and non-likers (based on the screening question, “How difficult do you find it to get your child to consume vegetables?” using a 9-point category scale, a child that scored ≤4 was categorised as a vegetable liker, a child that scored ≥5 was categorised as a non-liker). It was aimed to have a mix of girls and boys in each focus group. Food neophobia was measured using a 10-point neophobia scale (reported by parent), yielding a theoretical range between 10 (most neophillic) and 70 (most neophobic) [65].

Participants were provided with an AUD50 cash incentive for their participation. CSIRO’s Health and Medical Human Research Ethics Committee approved this study (approval 2019_083_LR). Written consent was obtained from parents and verbal assent was obtained from the children and recorded.

##### Focus Group Sessions

In total, eight focus groups of 45 min each were conducted. The duration was chosen to fit with the attention span of these young children [66,67]. Focus groups followed a structure outlined in an interview guide (see Supplementary Material Part 2), which was pilot tested. The focus group started with a personal introduction from the moderators, explanation of the purpose of the session, and instructions. After getting to know the children and their experiences with vegetables and foods in general, the 14 concepts were presented and discussed one by one. The researchers briefly explained what the concept entailed and then asked the group for their thoughts. The same questions were asked for each concept, including describing the positives and negatives of the concepts, and the most appropriate consumption situation.

Each focus group was led by two female moderators. One experienced moderator (JB) moderated all eight focus groups for consistency, and two others (MCB and JEH) moderated four sessions each. All three moderators had been involved in the development of the concepts and, therefore, knew which factors related to the CAMPOV model. The focus groups took place in a child-friendly focus room. Parents sat outside the room where children could see them through the glass wall. All focus groups were video recorded for analysis, and field notes were taken in case voices were not picked up by the camera.

##### Data Analysis

All recordings were viewed by at least two of the moderators together for content analysis, and were coded in Excel (Microsoft Office) to obtain immediate consensus on what was said verbally about, and what reactions were shown to, each concept. Codes used in the coding process related to the properties or the use of the specific concept (e.g., colour, shapes, flavours, eating experience). The first part of the conversation, which served as setting the context, was not coded or analysed. Positives and negatives, as well as overall comments for each concept, were recorded. Responses were then summarised by concept and interpreted by all three moderators together. Data saturation occurred after six focus groups, and the two last groups confirmed the findings. An independent *T*-test was used to test whether vegetable likers were less neophobic than vegetable non-likers, a *p*-value < 0.05 was considered statistically significant.

### 3.2. Results of Qualitative Evaluation of Concepts with Children

In total, 38 children participated in the focus groups, with a range of 4 to 6 children per focus group. Of these children, 20 (52.6%) were boys and 18 were (47.4%) girls. There was also a good distribution of age: 10 (26.3%) were 5 years old, 9 (23.7%) were 6 years old, 9 (23.7%) were 7 years old, and 10 (26.3%) were 8 years old. The average neophobia score of all children was 34.6 ± 9.5. Children who liked vegetables were less neophobic (31.2 ± 9.0) than children who disliked vegetables (37.3 ± 9.2), *p* = 0.047.

Table 3 presents an overall summary of results of the children’s evaluation of the concepts, with concepts grouped based on their relative interest. Children overall had a high interest in six of the concepts, which were the Rainbow Dippers, Ice cream and ice block, Fairy dust, the Rainbow Squeeze-mate, the Crunch & Sip KIT, and the Children’s Cooking KIT. Overall, these concepts seemed to be characterised by having high visual appeal, as well as a “fun” factor. Three concepts had medium interest overall, and children’s interest seemed partially related to their level of food neophobia. Children not interested in these concepts were either unfamiliar with or did not like the existing product on which the concept was based (e.g., nori sheets), or they had concerns about whether it would taste good. These two reasons, unfamiliarity, and concerns about good taste, seemed also to be the primary reasons for the five concepts that children were not interested in.

Full summaries of findings per concept can be found in Supplementary Table S2.

**Table 3:** Summary of children’s responses to concepts, with positives and negatives listed for each concept, shown with the overall interest in the concepts (high, medium, or low interest).

Overall Interest in Concept	Concept	Positives	Negatives
High	Rainbow Dippers	Visual appeal of coloured breadsticks and coloured dips, fun of dipping the food	Doubts about eating multiple flavours together in a rainbow dip
	Ice cream & ice block	Familiar with icy treats, visual appeal of colours	Worries about overpowering vegetable flavours
	Fairy dust	Visual appeal of vibrant colours, carrot flavour, extreme sourness was polarizing	Spicy powder was not liked
	Rainbow Squeeze-mate	Squeezing action was seen as fun, avoiding soggy bread was a benefit	Uncertainty of mixing of rainbow colours, dip should not be multiple flavours
	Crunch & Sip KIT	Mini vegetables were seen as cute, both mini-vegetables and cut-up vegetables were said to be easy to eat, variety of vegetables was appealing	Atypically coloured vegetables were not appealing for all children
	Children’s Cooking KIT	Cooking was considered to be a fun activity, when recipes are child-friendly	-
Medium	Vegetable sheets	Crispy texture and salty flavour were liked	Gooey in-mouth texture was not liked by children who do not like Nori sheets
	Pizza base	All children liked pizza and half found colours visually appealing	Doubtful about overpowering vegetable flavours, pizza should not change too much
	VeggieStix	Visual appeal of fun shapes, fun to eat from a stick	Vegetable-only sticks were least appealing
Low	Yoghurt with vegetables	Pink colour was appealing to most children	Concerns about overpowering vegetable flavour
	Poppables/VegOPop	Flavour and texture of popcorn is liked	Concerns about overpowering vegetable flavours
	Vegetable wraps & bread rolls	Visually appealing use of colours for half of children	Familiar product category should stay as is
	Veggie bites	Visually appealing fun shapes and bright colours	Vegetable filling instead of meat filling
	Sipp’a soup	Using a straw to sip soup is novel and fun	Soup is not a popular product to eat

## 4. Discussion

The current study proposed a new evidence-informed theoretical model to help guide the food industry with product development of vegetable-based products for children. An initial proof-of-concept evaluation showed support for several aspects of the model.

The focus group discussions using concepts provided very useful insights. Most of the factors in the CAMPOV model that were hypothesised to be able to positively influence children’s interest were found to contribute to children’s interests in the concepts. These factors were bright colours, fun shapes, bite-sized pieces, fun eating experience, good taste, imaginative language, familiarity, and role modelling. The factor for which we found no support was atypical colour (only some of the children were attracted to this factor). There was insufficient evidence for the role of texture (crunchiness and textural contrast) and specific taste profiles, in part because no actual tasting of concepts was involved in the focus groups. Although children showed ability to imagine the likely taste/flavour of several concepts, awareness of texture was low compared to awareness of other sensory modalities [68] and, therefore, likely not salient.

The proof-of-concept evaluation enhanced understanding of several factors. For example, children were interested in novel variations of products that already had high appeal, like pizza, but at the same time, some were concerned about changing the familiar flavour they liked. They expressed interest in multiple colours, but at the same time, were concerned that multiple colours may bring multiple flavours together, which they may not like. The concepts in which children were most interested all had a fun eating experience as part of the concept (e.g., dipping, sprinkling, squeezing, cooking themselves) or were considered to be a treat (e.g., ice blocks), and this element was specifically mentioned by children as appealing. Several of the concepts had associative learning opportunities as part of their design, which in this case referred to the opportunity to learn to like the flavour or texture of vegetables by being exposed to them as part of another product that they liked (e.g., learning to like the vegetables on which “Fairy dust” (a liked savoury powder) was sprinkled). This is an attribute that cannot be directly evaluated by children. Of the concepts that had those opportunities, some had high interest, whereas others did not, demonstrating that, in itself, it is not a critical success factor with children. In the current study, no actual tasting occurred, as concepts were paper-based. However, it was clear from the children’s responses that taste was a key priority for them, and they were raising concerns about the potential taste of some concepts, indicating that they were not interested if they did not taste good. In particular, children were worried about overpowering vegetable flavours.

Children’s social, cognitive, perceptual, and emotional development undergo enormous changes. Whereas the proposed model is aimed at younger children, there are large differences within this group that need to be considered. For example, there is good evidence on the role of cartoon characters in children’s vegetable acceptance [42,43,44,45,46]; however, the specific characters that may be successful for new products will differ with age and also over time. The level of food neophobia influences the degree to which children are open to new foods and, therefore, the more novel concepts may appeal more to neophilic children. Previous research also showed that the relative influence of appearance in vegetable categorisation decreased with age [10], therefore, the importance of extrinsic and intrinsic properties on acceptance may also change with age, which would need further investigation.

The product development model was informed by a literature review on intrinsic and extrinsic attributes in relation to children’s acceptance of vegetables. The proof-of-concept evaluation was based on concepts and undertaken via focus groups. Although it is a common first step in the initial stages of product development, this method has the limitation that no actual products were tasted. Further prototype development and quantitative sensory consumer acceptance testing with children is recommended to obtain further insights into children’s evaluation of the products, in the validity of the model, and to obtain further insights into the in-mouth properties (taste/flavour and texture). Previous research has demonstrated that the role of texture in fresh vegetables is difficult to establish because they co-vary in flavour and texture attributes simultaneously [11,30,36]; however, carefully designed experiments using vegetable-based products offer larger experimental control. Further research to determine the role of specific intrinsic and extrinsic attributes, as well as their interaction and their relative importance in children’s acceptance of vegetable-based products, is also recommended.

The current research can guide further sensory and consumer research to increase children’s acceptance of vegetable-based products, which will, in turn, provide further insights into the validity of the model. The model has potential for practical use by the food industry to support the development of new products for children with high sensory appeal, to increase vegetable intake amongst school-aged children. It should not be seen as a prescriptive model for successful product development, but rather as a framework that can help shape new concept ideas with high appeal for children. Although the main target audience for the model and the concepts is the food industry, some of the concepts and the model insights can also be applied in other settings, e.g., preparation by parents at home or by staff in school canteens. It is recommended to involve children and/or parents in further idea generation for new concepts through co-design research.

The different attributes in the model should be considered carefully when using them in different food categories. For example, modification of product properties may work out differently for novel snack items than in more familiar meal components or in cold versus hot products. Application by the food industry also needs to consider health and children’s development of food preferences. Firstly, although a sweet taste is a driver of liking, and saltiness can make products more palatable, the use of sugar and salt as condiments is detrimental to health. Therefore, delivery of these tastes should be achieved through use of core foods with such taste characteristics, such as sweet-tasting fruits or vegetables, or (salty-tasting) cheese. The combination of fruit and vegetable flavours was mentioned by many children in the focus groups as a promising strategy to mask strong vegetable flavours. Secondly, it is also important to consider that children’s food preferences are in large part shaped by their experiences with food [9,20,58] and, therefore, new vegetable-based products should not only appeal to children, but also need to take their sensory learning potential into account. Conducting sensory consumer acceptance studies over a longer period of time can provide useful insights into the dynamics of acceptance in order to optimally balance this aspect.

In conclusion, the children’s evaluation of new vegetable-based concepts has highlighted important attributes to consider in product development of new vegetable-based products for children. These attributes were bright colours, fun shapes, bite-sized pieces, fun eating experience, good taste, imaginative language, familiarity, and role modelling.

## Figures and Tables

**Table 1:** Children’s Acceptance Model for Product development Of Vegetables (CAMPOV model).

Factor	Properties to Promote Children’s Vegetable Acceptance
Intrinsic properties	
Appearance	Bright colours, atypical colours of veg, variety of colours, fun shapes, small sizes/bite sized
Taste/flavour	Sweet taste, suppression/absence of bitterness, good taste, pairing with liked tastes/flavours
Texture	Crunchiness, lack of textural contrast
Extrinsic properties	
Claims/branding	Sensory claims, imaginative language, fun characters on pack, absence of health claims
Psychological and situational factors	
Fun	Fun sensations, fun eating experience
Associative learning	Pairing with liked attributes
Previous experience	Encourage repeated trying, familiarity to existing
Role modelling	Mimicking parents, peers

**Table 2:** Concept name, description, and all potential appealing attributes from the literature and product mapping.

Concept Name	Concept Description	Potential Appealing Characteristics for Children
Rainbow Dippers	Combination of colourful dippers and dip	bright colours, atypical colours, variety (colour, flavour, texture), bite-sized, flavoursome, fun eating experience
Yoghurt with vegetables	Smooth, colourful yoghurt containing vegetable	bright colours, flavoursome, taste contrast, sourness, smooth texture
Ice cream & ice block	Ice cream and ice block containing vegetables	bright colours, atypical colours, colour variety, sweet taste, taste contrast (sweet/sour), flavoursome, novel sensation (sour taste), fun eating experience, familiarity with existing products
Vegetable sheets	Flat, crispy vegetable snacking sheets (like nori sheets)	bright colours, atypical colours, colour variety, flavoursome, novel sensation, crunchiness, fun eating experience, familiarity with existing products
Poppables/VegOPop	Crunchy vegetable popcorn	bright colours, atypical colours, colour variety, flavoursome, crunchiness, fun eating experience, familiarity with existing products
Pizza base	Pizza base with vegetable in the dough	bright colours, atypical colours, contrasts (colour, texture), flavoursome, fun eating experience
Vegetable wraps & bread rolls	Wraps and bread rolls with vegetable in the dough	bright colours, atypical colours, colour variety, fun shapes, familiarity with existing products
VeggieStix	A variety of single-bite veggies (raw or cooked) on a skewer	bright colours, fun shapes, contrasts (colour, shape, flavour, texture), flavoursome, fun eating experience, familiarity with existing products
Veggie bites	Vegetable-based bites, comes in nuggets or bite-sized patties	bright colours, atypical colours, fun shapes, shape contrast, bite-sized, textural contrast, fun eating experience
Sipp’a soup	Straw filled with vegetable-based powder with different flavours, used to sip soup through	flavoursome, novel flavour sensations, suppression of bitterness, fun eating experience, associative learning
Fairy dust	Vegetable-based powder with different flavours to sprinkle on vegetables and other food	bright colours, suppression of bitterness, taste contrast, novel flavour sensations (sour, spicy), fun eating experience, associative learning
Rainbow Squeeze-mate	Vegetable dip/sauce single-use dispenser	bright colours, atypical colours, contrast (colour, flavour), flavoursome, novel sensation, fun eating experience
Crunch & Sip KIT	Vegetable-based Crunch & Sip KIT with a medley of baby vegetables or cut-up vegetables, potentially with a subscription model	bright colours, atypical colours, fun shapes, contrasts (colour, flavour, texture), bite-sized, flavoursome, crunchiness, familiarity with existing eating occasion
Children’s Cooking KIT	Box that contains a child-friendly recipe with all ingredients in it to get children involved in cooking, potentially with a subscription model	fun shapes, taste and textural contrast, fun eating experience, role modelling (mimicking parents)

## Data Availability

The data are not publicly available as subjects could be re-identifiable due to the qualitative nature of the data.

## Supplementary Materials

The following are available online at https://www.mdpi.com/article/10.3390/foods11010096/s1, Part 1: Parent interview methodology and summary of findings (including Table S1 and Figure S1), Part 2: Focus group detail methodology and results, including concept description and full summaries of findings for each concept (including Table S2).

## References

[1] Calvert S.L. (2008). Children as consumers: Advertising and marketing. Future Child..

[2] Cairns G., Angus K., Hastings G., Caraher M. (2013). Systematic reviews of the evidence on the nature, extent and effects of food marketing to children. A retrospective summary. Appetite.

[3] Sadeghirad B., Duhaney T., Motaghipisheh S., Campbell N.R.C., Johnston B.C. (2016). Influence of unhealthy food and beverage marketing on children’s dietary intake and preference: A systematic review and meta-analysis of randomized trials. Obes. Rev..

[4] Beckerman J.P., Alike Q., Lovin E., Tamez M., Mattei J. (2017). The development and public health implications of food preferences in children. Front. Nutr..

[5] Poelman A.A.M., Delahunty C.M., de Graaf C. (2017). Vegetables and other core food groups: A comparison of key flavour and texture properties. Food Qual. Prefer..

[6] Mihrshahi S., Myton R., Partridge S.R., Esdaile E., Hardy L.L., Gale J. (2019). Sustained low consumption of fruit and vegetables in Australian children: Findings from the Australian National Health Surveys. Health Promot. J. Aust..

[7] Yngve A., Wolf A., Poortvliet E., Elmadfa I., Brug J., Ehrenblad B., Franchini B., Haraldsdóttir J., Krølner R., Maes L. (2005). Fruit and vegetable intake in a sample of 11-year-old children in 9 European countries: The Pro Children Cross-Sectional Survey. Ann. Nutr. Metab..

[8] Wootan M.G., Almy J., Ugalde M., Kaminski M. (2019). How Do Nutrition Guidelines Compare for Industry to Market Food and Beverage Products to Children? World Health Organization Nutrient Profile Standards versus the US Children’s Food and Beverage Advertising Initiative. Child. Obes..

[9] Köster E.P. (2009). Diversity in the determinants of food choice: A psychological perspective. Food Qual. Prefer..

[10] Zeinstra G.G., Koelen M., Kok F., de Graaf C. (2007). Cognitive development and children’s perceptions of fruit and vegetables; a qualitative study. Int. J. Behav. Nutr. Phys. Act..

[11] Baxter I.A., Schröder M.J.A., Bower J.A. (1999). The influence of socio-economic background on perceptions of vegetables among Scottish primary school children. Food Qual. Prefer..

[12] Poelman A.A.M., Delahunty C.M. (2011). The effect of preparation method and typicality of colour on children’s acceptance for vegetables. Food Qual. Prefer..

[13] Zampollo F., Kniffin K.M., Wansink B., Shimizu M. (2012). Food plating preferences of children: The importance of presentation on desire for diversity. Acta Paediatr..

[14] Olsen A., Ritz C., Kramer L., Møller P. (2012). Serving styles of raw snack vegetables. What do children want?. Appetite.

[15] Liem D.G., Russell C.G. (2019). Supersize me. Serving carrots whole versus diced influences children’s consumption. Food Qual. Prefer..

[16] Chung L.M.Y., Fong S.S.M. (2018). Appearance alteration of fruits and vegetables to increase their appeal to and consumption by school-age children: A pilot study. Health Psychol. Open.

[17] Zeinstra G.G., Koelen M.A., Kok F.J., de Graaf C. (2010). The influence of preparation method on children’s liking for vegetables. Food Qual. Prefer..

[18] Donadini G., Fumi M.D., Porretta S. (2012). Influence of preparation method on the hedonic response of preschoolers to raw, boiled or oven-baked vegetables. LWT Food Sci. Technol..

[19] Wardle J., Sanderson S., Gibson E.L., Rapoport L. (2001). Factor-analytic structure of food preferences in four-year-old children in the UK. Appetite.

[20] Mennella J.A. (2014). Ontogeny of taste preferences: Basic biology and implications for health. Am. J. Clin. Nutr..

[21] Mennella J.A., Reiter A.R., Daniels L.M. (2016). Vegetable and Fruit Acceptance during Infancy: Impact of Ontogeny, Genetics, and Early Experiences. Adv. Nutr. Int. Rev. J..

[22] Hill C., Wardle J., Cooke L. (2009). Adiposity is not associated with children’s reported liking for selected foods. Appetite.

[23] Breslin P.A.S., Beauchamp G.K. (1995). Suppression of bitterness by sodium: Variation among bitter taste stimuli. Chem. Senses.

[24] Bouhlal S., Issanchou S., Nicklaus S. (2011). The impact of salt, fat and sugar levels on toddler food intake. Br. J. Nutr..

[25] Van Stokkom V., Poelman A., de Graaf C., van Kooten O., Stieger M. (2018). Sweetness but not sourness enhancement increases acceptance of cucumber and green capsicum purees in children. Appetite.

[26] Hausner H., Olsen A., Møller P. (2012). Mere exposure and flavour–flavour learning increase 2–3 year-old children’s acceptance of a novel vegetable. Appetite.

[27] Bouhlal S., Issanchou S., Chabanet C., Nicklaus S. (2014). ‘Just a pinch of salt’. An experimental comparison of the effect of repeated exposure and flavor-flavor learning with salt or spice on vegetable acceptance in toddlers. Appetite.

[28] Baxter I.A., Schroder M.J.A. (1997). Vegetable consumption among Scottish children: A review of the determinants and proposed strategies to overcome low consumption. Br. Food J..

[29] Zeinstra G.G., Koelen M.A., Kok F.J., de Graaf C. (2009). Children’s hard-wired aversion to pure vegetable tastes. A ‘failed’ flavour-nutrient learning study. Appetite.

[30] Poelman A.A.M., Delahunty C.M., de Graaf C. (2013). Cooking time but not cooking method affects children’s acceptance of *Brassica* vegetables. Food Qual. Prefer..

[31] Baranowski T., Domel S., Gould R., Baranowski J., Leonard S., Treiber F., Mullis R. (1993). Increasing fruit and vegetable consumption among 4th and 5th grade students: Results from focus groups using reciprocal determinism. J. Nutr. Educ..

[32] Kirby S.D., Baranowski T., Reynolds K.D., Taylor G., Binkley D. (1995). Children’s fruit and vegetable intake: Socioeconomic, adult-child, regional, and urban-rural influences. J. Nutr. Educ..

[33] Savage J.S., Peterson J., Marini M., Bordi P.L., Birch L.L. (2013). The addition of a plain or herb-flavored reduced-fat dip is associated with improved preschoolers’ intake of vegetables. J. Acad. Nutr. Diet..

[34] Fisher J.O., Mennella J.A., Hughes S.O., Liu Y., Mendoza P.M., Patrick H. (2012). Offering “dip” promotes intake of a moderately-liked raw vegetable among preschoolers with genetic sensitivity to bitterness. J. Acad. Nutr. Diet..

[35] Carney E.M., Stein W.M., Reigh N.A., Gater F.M., Bakke A.J., Hayes J.E., Keller K.L. (2018). Increasing flavor variety with herbs and spices improves relative vegetable intake in children who are propylthiouracil (PROP) tasters relative to nontasters. Physiol. Behav..

[36] Baxter I.A., Jack F.R., Schröder M.J.A. (1998). The use of repertory grid method to elicit perceptual data from primary school children. Food Qual. Prefer..

[37] Poelman A.A.M., Delahunty C.M., de Graaf C. (2015). Vegetable preparation practices for 5–6 year old Australian children as reported by their parents; relationships with liking and consumption. Food Qual. Prefer..

[38] Szczesniak A.S. (1972). Consumer awareness of and attitudes to food texture II. Children and teenagers. J. Text. Stud..

[39] Reinaerts E., de Nooijer J., Candel M., de Vries N. (2007). Explaining school children’s fruit and vegetable consumption: The contributions of availability, accessibility, exposure, parental consumption and habit in addition to psychosocial factors. Appetite.

[40] Dazeley P., Houston-Price C. (2015). Exposure to foods’ non-taste sensory properties. A nursery intervention to increase children’s willingness to try fruit and vegetables. Appetite.

[41] Morizet D., Depezay L., Combris P., Picard D., Giboreau A. (2012). Effect of labeling on new vegetable dish acceptance in preadolescent children. Appetite.

[42] Kraak V.I., Story M. (2015). Influence of food companies’ brand mascots and entertainment companies’ cartoon media characters on children’s diet and health: A systematic review and research needs. Obes. Rev..

[43] Karpyn A., Allen M., Marks S., Filion N., Humphrey D., Ye A., May H., Gardner M.P. (2017). Pairing animal cartoon characters with produce stimulates selection among child zoo visitors. Health Educ. Behav..

[44] Pempek T.A., Calvert S.L. (2009). Tipping the balance: Use of advergames to promote consumption of nutritious foods and beverages by low-income African American children. Arch. Pediatr. Adolesc. Med..

[45] Nicklas T.A., Goh E.T., Goodell L.S., Acuff D.S., Reiher R., Buday R., Ottenbacher A. (2011). Impact of commercials on food preferences of low-income, minority preschoolers. J. Nutr. Educ. Behav..

[46] Hanks A.S., Just D.R., Brumberg A. (2016). Marketing vegetables in elementary school cafeterias to increase uptake. Pediatrics.

[47] Leonard B., Campbell M.C., Manning K.C. (2019). Kids, caregivers, and cartoons: The impact of licensed characters on food choices and consumption. J. Public Policy Mark..

[48] Charry K.M. (2014). Product placement and the promotion of healthy food to pre-adolescents: When popular TV series make carrots look cool. Int. J. Advert..

[49] Lagomarsino M., Suggs L.S. (2018). Choosing imagery in advertising healthy food to children: Are cartoons the most effective visual strategy?. J. Advert. Res..

[50] Keller K.L., Kuilema L.G., Lee N., Yoon J., Mascaro B., Combes A.-L., Deutsch B., Sorte K., Halford J.C. (2012). The impact of food branding on children’s eating behavior and obesity. Physiol. Behav..

[51] Thapa J.R., Lyford C.P. (2018). Nudges to increase fruits and vegetables consumption: Results from a field experiment. J. Child Nutr. Manag..

[52] Byrne E., Nitzke S. (2002). Preschool children’s acceptance of a novel vegetable following exposure to messages in a storybook. J. Nutr. Educ. Behav..

[53] Owen L.H., Kennedy O.B., Hill C., Houston-Price C. (2018). Peas, please! Food familiarization through picture books helps parents introduce vegetables into preschoolers’ diets. Appetite.

[54] Wardle J., Huon G. (2000). An experimental investigation of the influence of health information on children’s taste preferences. Health Educ. Res..

[55] Hémar-Nicolas V., Hapsari H.P., Angka S., Olsen A. (2021). How cartoon characters and claims influence children’s attitude towards a snack vegetable–An explorative cross-cultural comparison between Indonesia and Denmark. Food Qual. Prefer..

[56] Steiner J.E. (1979). Human facial expressions in response to taste and smell stimulation. Adv. Child Dev. Behav..

[57] Laureati M., Sandvik P., Almli V.L., Sandell M., Zeinstra G., Methven L., Wallner M., Jilani H., Alfaro B., Proserpio C. (2020). Individual differences in texture preferences among European children: Development and validation of the Child Food Texture Preference Questionnaire (CFTPQ). Food Qual. Prefer..

[58] Bell L.K., Gardner C., Tian E.J., Cochet-Broch M.O., Poelman A.A., Cox D.N., Nicklaus S., Matvienko-Sikar K., Daniels L.A., Kumar S. (2021). Supporting strategies for enhancing vegetable liking in the early years of life: An umbrella review of systematic reviews. Am. J. Clin. Nutr..

[59] Appleton K.M., Hemingway A., Saulais L., Dinnella C., Monteleone E., Depezay L., Morizet D., Perez-Cueto F.A., Bevan A., Hartwell H. (2016). Increasing vegetable intakes: Rationale and systematic review of published interventions. Eur. J. Nutr..

[60] Appleton K.M., Hemingway A., Rajska J., Hartwell H. (2018). Repeated exposure and conditioning strategies for increasing vegetable liking and intake: Systematic review and meta-analyses of the published literature. Am. J. Clin. Nutr..

[61] Holley C.E., Farrow C., Haycraft E. (2017). A systematic review of methods for increasing vegetable consumption in early childhood. Curr. Nutr. Rep..

[62] Blissett J., Fogel A. (2013). Intrinsic and extrinsic influences on children’s acceptance of new foods. Physiol. Behav..

[63] Morgan M., Gibbs S., Maxwell K., Britten N. (2002). Hearing children’s voices: Methodological issues in conducting focus groups with children aged 7–11 years. Qual. Res..

[64] Kelly L. (2013). Conducting focus groups with child participants. Dev. Pract. Child Youth Fam. Work J..

[65] Pliner P. (1994). Development of measures of food neophobia in children. Appetite.

[66] Gibson F. (2007). Conducting focus groups with children and young people: Strategies for success. J. Res. Nurs..

[67] Popper R., Kroll J.J. (2005). Conducting sensory research with children. J. Sens. Stud..

[68] Szczesniak A.S. (2002). Texture is a sensory property. Food Qual. Prefer..

