# Supplementary material for: Development and Proof-of-Concept Evaluation of a Sensory Science-Based Model for Product Development of Vegetable-Based Products for Children
Source: Foods. 2021 Dec 30;11(1):96. doi: 10.3390/foods11010096 (PMC8750587; doi:10.3390/foods11010096)
Supplement: Supplementary file 1 [file foods-11-00096-s001.zip › foods-1493118-SI.pdf]

Supplementary material for:
Development and proof-of-concept evaluation of a sensory science-based model for product development of vegetable-based products for children.

Part 1: Qualitative evaluation (interviews) of concepts by parents

Parents purchase most food for their children, therefore gathering insights on parental attitudes towards vegetable-based concepts for children is important. The aim of this sub-task of the study was to gather qualitative information from parents on 24 newly developed vegetable-based concepts. A qualitative study was undertaken by conducting fifteen one-on-one semi-structured interviews with parents of 5-to 8-year-old children.

Material and methods

Participants

English speaking parents of 5-8-year-old children were invited to participate in the study through newsletters from primary schools and sport associations in the Greater Sydney area, with suburbs representing a spread across high, medium and low socio-economic status (SES). Participants were given a $40 gift card as incentive to participate. CSIRO's Low Risk Review Panel ethics committee approved this study and informed consent was gathered from all participants.

Data collection and analysis

Fifteen one-on-one semi-structured interviews (maximum duration of 60 minutes) were conducted between August and October 2019. During these interviews, 24 product concepts were discussed using a written interview guide. Multiple (2-4) concepts were presented together by providing participants with the A4 presentation designs. This was done to allow products for similar occasions to be discussed simultaneously to extract some information about the eating opportunity (e.g. vegetable pizza base and vegetable pasta and noodles are both concepts relating to dinner components). The concepts were presented to parents in the order as listed in Table S1. Each concept was presented on an A4 sheet and showed the concept name, a product description, visual representation of the concept (photograph) as well as defining characteristics (for example see Figure S1). Parents were provided with the A4 presentation design and read the information themselves and provided their spontaneous reactions to the concept. They were then asked to provide their opinions on the positives and negatives of several intrinsic and extrinsic aspects, as well as the most appropriate setting(s) (home, school, etc.) and eating occasion(s) for the concepts. The interviewer prompted parents with questions to elicit other information on the concepts. To facilitate the roll-out of the interviews, three concepts (soup experience enhancers) were grouped on one slide and as such, 22 concept slides were presented to parents (see Table S1 for the list of concepts that were tested).

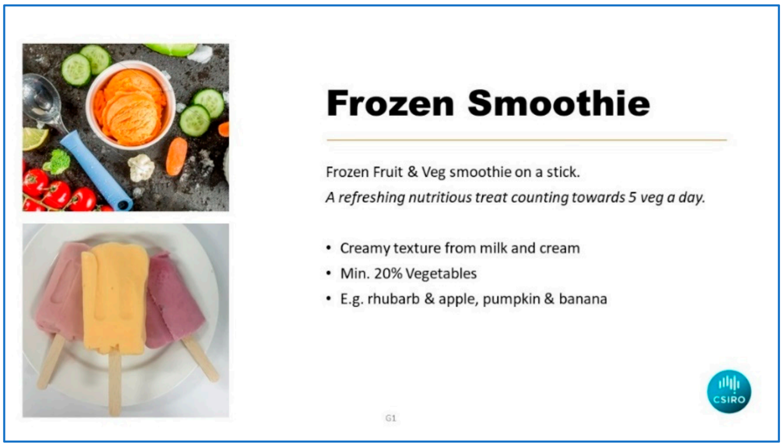

Figure S1. Example of concept as presented to parents.

Table S1. Concepts as tested with parents (n = 24).

Number	Concept	Description and textual presentation of the concepts
1	Vegetable kebabs	A variety of single bite veggies on a skewer.<br>*A fun and convenient way to eat a rainbow.*<br>Comes in these forms:<br>• Raw vegetables kebab<br>• Grilled vegetables kebab<br>• Sandwich kebab
2	Stuffed baby capsicum	Baby capsicums stuffed with any kind of filling.<br>*Perfect as a snack or in the lunchbox.*<br>• Variety of fillings e.g. coleslaw, beetroot, humus, couscous<br>• Consumed raw or baked in the oven
3	Crunch & Sip KIT	Vegetable based Crunch & Sip KIT.<br>*Save time with a premade Crunch & Sip KIT.*<br>Choice of:<br>• Mix of whole baby vegetables<br>• Medleys of freshly cut vegetables<br>• Freshly pureed vegetable soup (50-80% vegetables)<br>*Also available as a subscription at school.*
4	Vegetable dippers	Mini sticks with a dip.<br>*They are perfect as a snack for in the lunchbox or on the go!*<br>• Dip: vegetable-enriched dip or non-vegetable dip (e.g. cheese) or a combo "Rainbow" dip<br>• Dippers: breadsticks (35% vegetable) or fresh vegetables (e.g. carrot) or rice cakes (35% vegetable)
5	Seasoning sachets	Spice up your food with these exciting seasonings.<br>*Dip in or sprinkle of top for new flavour sensations.*<br>• Made from vegetables<br>• Different flavours: sweet & sour, savoury/umami (with vegetable powder)
6	Vegetable pizza base	A pizza base like any other but with vegetables baked in the dough; an easy and tasty way to eat more vegetables!<br>*Kids can top their own pizza with whatever they like and enjoy it during lunch or dinner.*<br>• Min 60% vegetables (e.g. cauliflower)<br>• Cauliflower of different colours for more fun
7	Vegetable pasta & noodles	100% vegetable pasta or noodles to use in your regular recipes: more vegetables in 1 meal.<br>*Simply steam in the microwave and serve with a sauce and extra veggies!*<br>• Natural vegetable colours<br>• Replace carbs with vegetables
8	Mini veggie burgers	Mini vegetable patties.<br>*A fun choice for Meatless Mondays.*<br>• Meat-free<br>• Loaded with vegetables<br>• Comes in different flavours, shapes and natural colours for variety<br>• Mini size for little mouths

9	Vegetable based wraps & bread rolls	Wraps and bread rolls with vegetables incorporates in the dough.<br>*An easy way to eat more vegetables!*<br>• 35% vegetable (e.g. spinach, beetroot)<br>• Eat them for lunch or dinner<br>• Variety of natural vegetable colours
10	Squeeze-mate	Vegetable spread in a 'squeeze on' package.<br>*Squeeze it on a sandwich for lunch and avoid soggy bread.*<br>• More vegetables than regular squeeze-on sauces<br>• Different flavours: 1 flavour or multiple flavours in 1 package
11	Vegetable sushi	Ready-to-eat sushi with veggie rice (e.g. broccoli or cauliflower); an easy and tasty way to eat more vegetables!<br>*Perfect for lunch or as a snack.*<br>• Min. 80% vegetables<br>• Variety of regular sushi fillings, like seafood and/or vegetables
12	Vegetarian steamed buns	Ready-to-eat steamed buns with vegetable incorporated in the dough and a vegetarian filling.<br>*Steam in the microwave and serve with a sauce for lunch.*<br>• Variety of natural vegetable colours<br>• Variety of fillings<br>• Balanced meal on the go
13	Unicorn slime & Dragon spit	A yoghurt containing vegetables.<br>*A tasty product feeding your imagination.*<br>• 10-40% vegetables<br>• Tasty flavour combinations (e.g. rhubarb/apple, carrot/ginger)<br>• Contains diverse bacteria from yoghurt
14	Sweet treats with hidden vegetables	A range of sweet treats with added vegetables.<br>*Healthier alternative than regular treats.*<br>• Min. 20% vegetables<br>• E.g. pumpkin/sweet potato brownie, avocado mousse, zucchini-carrot cake<br>• High in fibre, low in sugar
15	Veggie snack bar	A savoury, tasty, chewy bar with crunchy bits.<br>*Healthier alternative than regular snack bars.*<br>• Loaded with vegetables (between 12-50%) and grains/nuts<br>• Snack on the go<br>• Variety of flavours (e.g. BBQ, honey soy chicken)
16	Vegetable sheets	A crispy, light and tasty snack made of real vegetable fibres.<br>*Easy on the go; alternative to nori sheets or potato chips.*<br>• Nutrient dense (min. 85% vegetables)<br>• Natural vegetable colours and flavours<br>• Bite-sized pieces
17	Veggie bliss balls	Soft and chewy bite-sized veggie Bliss Balls.<br>*A perfect vegetable-based snack.*<br>• A range of vegetable bases (e.g. carrot, spinach)<br>• Naturally sweetened (e.g. date or pumpkin)<br>• Gluten-free
18	Frozen smoothie	Frozen fruit & veg smoothie on a stick.

		A refreshing nutritious treat counting towards 5 veg a day.<br>• Creamy texture from milk and cream<br>• Min. 20% vegetables<br>• E.g. Rhubarb & apple, pumpkin & banana
19	Ice block	Frozen fruit & veg on a stick.<br>*A refreshing treat counting towards 5 veg a day.*<br>• Tangy and sweet, dairy-free<br>• Min. 20% vegetables<br>• E.g. spinach & lemon, carrot & ginger
20	Gazpacho Pouch	A pouch of vegetable soup.<br>*Sip straight from the pouch.*<br>• 50-80% vegetables<br>• Smooth texture without chunks
21-23	Soup experience enhancers	Enhance your vegetable soup experience.<br>• Nutrition: Top your soup with roasted chickpeas and make it a balanced meal.<br>• Fun: Savoury filled cracker to dip or sip your soup.<br>• Sensation: A flavoured straw to sip your soup for an exciting flavour sensation (e.g. coconut, chilli, bacon, cheese).
24	Children's Cooking KIT	All the ingredients for a delicious dish in 1 KIT.<br>*Easy and fun way to get your child cooking!*<br>• Lots of fresh vegetables<br>• Builds children's independence and cooking skills<br>• Fun and easy recipes specifically made for kids

Each interview was audio recorded and transcribed verbatim. In accordance with the Consolidated criteria for reporting qualitative research (COREQ) [69], the transcript was then reviewed by the interviewee to ensure their responses were correctly transcribed. Transcripts were then coded independently by two researchers with the help of MAXQDA software (VERBI Software GmbH) until they reached consensus. Codes were relating to specific product characteristics (e.g. colour, shape, flavours, fun). After the coding process was finished, responses for each concept were analysed.

Main findings

Parents were most positive about the Vegetable pizza base, Vegetable dippers, Crunch & Sip KIT, Vegetable kebabs, Children's Cooking KIT, Flavoured straws, Frozen smoothie and Ice block. Reasons for these preferences were appealing colour, taste, texture and shapes for children, their child's familiarity with the concept, ease to consume finger foods, a fun way of consuming (like dipping, eating from a stick and sipping through a straw), and the fun aspect of children helping with cooking. According to the parents, atypical colours could either be an advantage or also put children off when it came to very familiar concepts (e.g. pizza base and sushi). Their rationale was that you shouldn't change too much about a popular dish because children will doubt the taste if it looks different than what they expect it to look like. They were, furthermore, wondering if bright colours were a result of additives and if they were that would be a barrier for them to buy these products because they said to try to limit using foods with artificial colourants and flavours. Parents' attitudes were the least positive towards the Gazpacho pouch, because of the anticipated taste and they were not familiar with cold soups and assumed their child would also not be keen to try it due to unfamiliarity; the Stuffed baby capsicum, because of the capsicum flavour that seemed polarizing (some parents said their child doesn't mind capsicum, others said their child does not like the taste of capsicum at all) and messiness while consuming; and the Seasoning sachet, because they felt it was very processed and associated it with artificial flavours which is not in line with their beliefs that it is healthier to avoid processed foods.

Regarding the setting in which concepts could become available, parents were most positive about offering the new vegetable-containing food products for use at school (e.g. via the school canteen) rather than at home, because they were concerned about the use of single-use plastic and costs.

Part 2:Focus Group Evaluation

Materials and methods

Recruitment:

Parents of prospective participants were invited per email to complete an online screening survey to determine if their child was eligible to participate (please note these were not the parents that participated in the parent interviews). Children needed to live in the Greater Sydney area and needed to be able to attend the focus group in the research facility. Children were excluded when they had known food allergies or when their parent indicated they found it very difficult to get their child to consume vegetables (based on the screening question "How difficult do you find it to get your child to consume vegetables?" Answers ranged from 'Not difficult at all' = 1 to 'Very difficult' = 9; children scoring a 9 were excluded). As part of the screening questionnaire, parents also completed a Neophobia scale for children [65]. This scale consists of 10 questions and each question is rated on a scale from 1 – 7 (disagree strongly to agree strongly). A sum score ('Neophobia score') is calculated (with several items requiring reverse coding) which ranges between 10 and 70. The higher the Neophobia score, the more neophobic a child is.

Focus group structure:

The focus group started with a personal introduction from the moderators (names and background), explanation on the purpose of the session and instructions ("listen and be respectful to each other", "there are no right or wrong answers, every opinion is important and it's fine when opinions differ", etc.) questions to set the context ("what vegetables do you like to eat?", "do you help with grocery shopping?", "what do you put in your lunchbox for school?"). After getting to know the children and their experience with vegetables and foods in general, the 14 concepts were presented and discussed one-by-one in the order as shown in Table 1 in the main text.

When discussing the concepts, the researchers briefly explained what the concept entailed and then asked the group for their thoughts. For each concept the same questions were asked (see full interview guide below). For some concepts, extra questions were prepared that focused on specific concept properties and were used to prompt the children when needed.

The presentation of a few concepts was also supported by using props: Cheese dippers (The Laughing Cow) for the Rainbow dippers, a tomato sauce squeeze package (MasterFoods) for the Rainbow Squeeze-mate, nori sheets (Ceres Organic) for the vegetable sheets, and a pre-prepared example of a Crunch & Sip KIT with baby vegetables.

Full interview guide:

1. **Introduction part:**
   a. Introduce yourself (make it a bit fun) and let kids mention their names
   b. Introduce the objective of the conversation:

   "We're going to show you some pictures of new food ideas with vegetables today which don't exist yet, and discuss these as a group. We would like to know what you think of those ideas. We would like to hear what you like about it and what you don't like about it so we can make foods that you and your friends would like to eat. If you have some other ideas yourselves, you can share these with the whole group."

"We will also record this with the camera (point at it) to listen back to the video afterwards to make sure we have all the information. Your mums/dads will be sitting just over there the whole time."

c. Ask for verbal assent:

"Are you happy to take part?" (each child needs to confirm with 'yes')

d. Instruction on behaviour:

"We will discuss these product ideas as a group, so try to listen to each other, speak one at a time, and everyone is entitled to have their own opinion, so we'll be polite to each other and don't be rude to each other. OK?"

2. **Questions to get more insights into the context of eating habits (don't need to ask all of these):**
    a. Can you tell me a bit about who shops for food and cooks at home? Do you help?
    b. When you go to school, do you take a lunchbox? Do you help with making the lunchbox or can you choose what goes in the lunchbox? And do you sometimes buy food from canteen at school? What do you like to eat when you are at school?
    c. When you come home from school, do you get an afternoon tea snack? What kind of snacks?
    d. Do you ever get a snack in the car when you're on your way to sports? What kind of snacks do you eat then?
    e. Can you tell me which vegetables you see here in front of you? You can eat them if you like to (point at tasting platter).
    f. Do you like eating vegetables? When do you eat vegetables usually?
3. **Show first concept and explain it**
4. **Ask general concept questions:**
    a. "What do you think about this product?"
    b. "What do you like about this?"
    c. "What don't you like about it?"
    d. "What part of it do you like the most? Why?"
    e. "If it was bought for you would you like to eat it?"
5. **Add specific questions per concept, see quiz cards**
6. **Setting questions:**
    a. "Where would you like to eat it? At home or at school? Or maybe when you go to somewhere in the car?"
    b. "Why there [location mentioned]?"
    c. "And when would you like to eat it? As breakfast, or as lunch, or as dinner? Or maybe a snack?"
    d. "Why then [meal moment mentioned]?"
7. **Summarize what has been said about this concept and ask:**
    a. "Did you want to add anything else about this one before we go to the next product?"
8. **Show next concept, repeat steps 3 – 7.**
9. **Wrap-up:**
    a. Final comments: "Do you have anything you would like to add that we haven't discussed yet, or do you have any cool ideas for products?"
    b. Closing: "Thanks for your help today!"

Focus Group Evaluation Results section

Table S2. Concept name, description and full summaries of findings for each concept.

Concept name and description	Summary of results
**Rainbow dippers:** Combination of colourful dippers and dip	Children were presented with 'Cheese dippers' (The Laughing Cow). Most children were familiar with these and said they liked the dipping way of eating. Overall, most children were interested in the Rainbow Dippers. For the dippers, bread sticks and carrot sticks were indicated as most interesting. The children liked the different coloured bread sticks more than the plain bread sticks. They didn't recognise what the rice cakes were. For the dips, overall, the multi-coloured (rainbow) dip was liked more from a visual perspective than the single colour dip. However, children were unsure about whether they would like multiple flavours of dip at the same time. The cheese dip was less liked than the rainbow or the avocado dip. The following flavours were proposed for the dips by the children: avocado, carrot, capsicum, hummus and tomato. Children said they could see themselves eating this at school.
**Yoghurt with vegetables:** Smooth colourful yoghurt containing vegetable	Most children liked yoghurt but associated it with flavours as vanilla, strawberry and mango. Most were a bit unsure about this concept, as they found it hard to comprehend what a vegetable flavoured yoghurt would taste like. There was overall agreement that the pink yoghurt (see presentation) was interesting, but flavour was more important; it should taste good and not too much like vegetables. Most children liked the prompted idea of a combination of fruit and vegetables better than solely vegetable flavours, mentioning the fruit flavour might mask the vegetable flavour. Besides the pink yoghurt, there were mixed responses to other presented colours; some liked the bright colours, while others had issues with the green and orange yoghurts. Despite this, carrot and cucumber were mentioned as acceptable flavours. Children were divided on the pouch packaging; sometimes mentioned as being for babies (across all age groups), while others mentioned it was convenient for eating and playing at the same time during the lunch break. Children were divided whether they would eat it at home or school indicating that it would depend on whether it came in a pouch or not.
**Ice cream & ice block:** Ice cream and ice block containing vegetables	Ice cream and ice blocks are very familiar to children; they said they often eat ice blocks at home or at school and they like to eat ice cream at home. Vegetable-only flavours were not well received, but most children were open to the idea of a combination of fruit and vegetable flavours, specifically for ice blocks. This is likely because the sweet and sour taste combination might mask the less liked vegetable flavours. Extreme sour tasting ice blocks were polarising; some children liked sourness, others strongly disliked the idea. Children liked the presented carrot ice cream, both in terms of colour and expected flavour. All children liked bright colours in ice blocks and ice cream, especially green and pink. Children mentioned that the following vegetable flavours could work for ice blocks: beetroot, tomato, carrot and cucumber. Most children said to be more likely to eat them at home, but the canteen might still be an option as they said to already eat ice blocks there.
**Vegetable sheets:** Flat, crispy vegetable snacking sheets (like nori sheets)	Children were shown, and could taste, nori sheets to improve comprehension of the concept. It was found that nori sheets were polarising. When asked what children liked about them, they mentioned the saltiness. The texture in mouth, turning from crispy to gooey, was also found to be polarising; everyone liked the initial crispiness but only a couple liked the gooeyness in mouth. Responses were mixed about the idea of this type of thin crispy sheets with vegetable flavours;

	children that liked the seaweed flavour were not really open to the idea of a vegetable flavour because it was already good as is, while children that didn't like the seaweed flavour were more open to vegetable flavoured sheets. The following vegetable flavours were suggested: carrot, capsicum, tomato, cucumber, beetroot, broccoli and salty corn. Most children liked the different colours, but the shown sushi with different coloured outsides gave mixed reactions; some preferred the bright colours, others preferred normal looking sushi rolls. There were no strong opinions on where to eat the vegetable sheets, some children would like to eat it at school, others suggested to have it as a snack on the go.
**Poppables / VegOPop:**<br>Crunchy vegetable popcorn	Almost all children liked popcorn, but the idea of having multi coloured popcorn (concept presentation) yielded hesitancy. When it was explained how multi-coloured corn could be grown on a farm, most were excited about the idea. Then the popped vegetable idea was explained, and most children said to like the popped texture but said it should taste like popcorn, not another vegetable. Popped broccoli or kale wasn't appealing because of the green colour and the strong vegetable flavours. The names 'Poppables' and 'VegOPop' were proposed, and it was asked whether the children liked those names, and if they preferred to have an indication of the vegetable in it (e.g., BrocOPop for broccoli). The name Poppables wasn't liked much, VegOPop was slightly better accepted. Vegetable non-likers more often indicated they liked a name to tell them which vegetable flavour it is so they know what they are getting, while vegetable likers were not fussed about not knowing what flavour they were getting.
**Pizza base:**<br>Pizza base with vegetable in the dough	Most children liked eating pizza and were open to the idea of a pizza base with vegetables mixed into the dough. The different colours were appealing, especially to vegetable likers. They were also more open to the idea of a pizza base with vegetable flavour, while non-likers preferred a normal looking and tasting base as they worried the base with vegetables would taste different from their expectation. The purple base was appealing to many children, but there were doubts raised about its flavour if it was beetroot. The coloured cauliflowers caused more negativity than curiosity; almost no one was interested in a purple or orange cauliflower pizza base. Children mentioned carrot, tomato and sweet potato as potential flavours, both from a colour and flavour perspective. When an option of a 100% vegetable base was described, almost none of the children were interested due to taste concerns, they mentioned that it should be mostly dough but could have a bit of vegetables in it.
**Vegetable wraps & bread rolls:**<br>Wraps and bread rolls with vegetable in the dough	Most children said they like to eat burgers and/or wraps. When presented with the vegetable-containing, coloured, wraps and bread rolls, only half of the children found the colours appealing, the other half preferred them in a plain colour. The children that liked the colours of the bread rolls and wraps seemed to prefer the brighter colours over the duller colours. It seemed that most children preferred the bread rolls and wraps to taste like normal bread products, i.e., with not too much vegetable flavour in them.
**VeggieStix:**<br>A variety of single bite veggies (raw or cooked) on a skewer	Most children had eaten from a stick before, mostly meat kebabs and some had fruit kebabs before, only for a few it was a novel idea to eat from a stick. Most children said they think it's fun to eat from a stick and were interested in eating the VeggieStix. The sandwich-style stick was preferred over the vegetable-only sticks by most children. The reasons they preferred this concept was the inclusion of different foods such as bread, cheese and ham, as well as vegetables, and because it came with nice shapes. When comparing the two vegetable-only sticks, most children seemed to prefer the raw vegetables over the cooked vegetables, they couldn't provide detailed reasons for their preference except for preferring raw vegetables in general. When talking about where they would eat it, some

	children mentioned that they might not be allowed to bring food on a stick to school for safety reasons and therefore saw themselves eating this at home and not in school.
**Veggie bites:**<br>Vegetabled-based bites, comes in nuggets or bite sized patties	Overall, children said they like to eat chicken nuggets; however, most children seemed to prefer 'normal' meat / chicken nuggets to vegetable-filled bites. Many children had difficulties comprehending the idea of the vegetable fillings and what the Veggie Bites would taste like. Looking at the concept presentation, most children found the different shapes (upper left picture) appealing, as was the bright coloured filling on the bottom pictures. Some children mentioned they would like to have the coloured filling, but it should mainly taste like meat. When given the option of a 'surprise-filling' (i.e.: not knowing what vegetable-flavoured filling was inside), the vegetable likers were more open to the idea than the vegetable non-likers who were opposed to not knowing what vegetable filling they would get. The bites weren't really mentioned as being part of a meal, but as a stand-alone product like chicken nuggets; they could see themselves eat these at school and at home as snacks/lunch, not for dinner.
**Sipp'a soup:**<br>Straw filled with vegetable-based powder with different flavours, used to sip soup through	Some children knew the "Sippah straws" but most had difficulty making the connection to drinking soup through a flavoured straw. After clarifying the concept, such as "the straw would only be for smooth soups without chunks", children could imagine liking a straw with bacon flavour, cheese flavour or carrot flavour. Soup was not consumed that often by most children, especially not in a school setting, however, children with an Asian background mentioned that they ate soup regularly, but these were mostly soups with noodles and chunks of vegetables and/or meat in them and they felt the straw could not be used when eating these types of soups.
**Fairy dust:**<br>Vegetable-based powder with different flavours to sprinkle on vegetables and other food	Overall, most children seemed open to this concept of either dipping their vegetables in the 'fairy dust' or sprinkling 'dust' on top of their vegetables as both are actions most of them like to do. Some children were concerned the 'fairy dust' would not stick on the vegetable and fall off. A few children suggested to overcome this by combining the 'dust' with a stickier component, for example served with the vegetable yoghurt or rainbow dip concepts. Most children found the bright colours of the Fairy dust appealing. Carrot flavoured dust was mentioned a few times as being a good flavour. Some children liked the sour taste idea; however, this was polarising because not all children liked a sour taste. A spicy chilli flavoured dust was not appealing to the large majority as they said they didn't like spicy foods.
**Rainbow Squeeze-mate:**<br>Vegetable dip/sauce single use dispenser	To make sure the children would understand the concept, we showed them a 'squeeze-on' container with tomato sauce from MasterFoods. All children knew these squeeze-on containers and liked using them, because they said it was fun to do. However, they associated them with eating pies and sausage rolls. After explaining that you could put the contents on bread or crackers, most of the children liked the idea of doing that during lunch. Most children liked the look of the rainbow spread with the different and vibrant colours; however, some worried the colours would mix and that the mix becomes unappealing. Furthermore, children were doubtful about the rainbow spread having multiple flavours, some children said they preferred multiple colours but only one vegetable flavour. Vegetable flavours that they mentioned as appealing were: avocado, tomato, beetroot, cucumber, carrot, pumpkin.
**Crunch & Sip KIT:**<br>Vegetable-based Crunch & Sip KIT with a medley of baby vegetables or cut-up	Most children had a Crunch & Sip break at school and told it was only a short moment (about 5 minutes) in the morning during which they usually ate fruit and sometimes vegetables. Most children liked the Crunch & Sip KIT idea and both the mini/baby vegetables, and the cut-up vegetables were liked a lot. For the

vegetables, potentially with a subscription model	mini/baby vegetables, several positives were mentioned including cuteness, ability to grab and hold with one hand and they are easy to eat. However, they thought that cut-up vegetables were even easier to eat. For both ideas they liked the mix of colours and the variety of vegetables. Including atypically coloured vegetables was also tested and some children liked the idea of different coloured carrots and tomatoes if they tasted as expected. Most children were not so keen on trying a tomato looking like a tiny watermelon. Overall, children liked the proposed idea of getting a Crunch & Sip KIT subscription from the canteen.
**Children's Cooking KIT:** Box that contains a child-friendly recipe with all ingredients in it to get children involved in cooking, potentially with a subscription model	Instantly, all children loved the Cooking KIT idea, including getting a chef's hat, as they said that they like to help out with cooking at home. No negatives were mentioned about this concept. The children were interested in making the following dishes: small snacks, pizza, pasta dishes, nachos, fish & chips, fruit salad, lasagne and soup.
